# The effect of antibacterial mouthwash on blood pressure in humans: a scoping review

**DOI:** 10.1186/s12872-026-06572-4

**Published:** 2026-09-16

**Authors:** Bhavik Singh, Parker North, Zoë M. Rushetsky, Richard C. Brito, Francis E. Jenney

**Affiliations:** 1 Philadelphia College of Osteopathic Medicine, Suwanee, GA USA; 2 University of Florida College of Medicine, Gainesville, FL USA; 3 College of Pharmacy & Pharmaceutical Sciences, Institute of Public Health, Florida Agricultural & Mechanical University, Tallahassee, FL USA

**Keywords:** Mouthwash, Blood pressure, Hypertension, Antibacterial

## Abstract

**Background:**

Oral hygiene is a key component of health. Practices for oral hygiene include brushing teeth with toothpaste, flossing, and mouth washing. It has been reported that 36% of United States adults use mouthwash daily. However, antibacterial mouthwashes kill oral nitrate-reducing bacteria, thus impairing nitric oxide production. Nitric oxide is a key molecule in vasodilation and blood pressure regulation, often acting as a mediator, so there is a potential for antibacterial mouthwashes to cause elevated blood pressure. Therefore, we conducted a scoping review to address the question: Does antibacterial mouthwash use significantly increase blood pressure in humans?

**Methods:**

A scoping review was conducted utilizing the databases PubMed and Scopus. Our search terms included “mouthwash,” “chlorhexidine,” “antibacterial,” “salivary nitrite,” “nitrate,” and MeSH terms like “blood pressure” and “hypertension.” Inclusion criteria required primary research articles since 2014 that analyzed changes in blood pressure in human adults after using antibacterial mouthwash. Studies with pediatric participants, animal studies, reviews, or studies that did not measure blood pressure changes were excluded. 51 studies were retrieved from the search, and after independent screening by two reviewers, nine studies were included in the scoping review. Reviewers extracted data on study methodology, sample size, participant characteristics, type of mouthwash, mouthwash regimen, and study results. Reviewers also appraised study quality using a modified Downs and Black assessment checklist.

**Results:**

Nine studies were included: four randomized crossover trials, three nonrandomized crossover trials, one active-controlled trial, and one observational study. The studies were of good quality (mean modified Downs and Black score was 21.9 ± 2.9 out of 28 points). Five studies found significant blood pressure elevations after mouthwash usage, while four studies found no significant relationship.

**Conclusions:**

Heterogeneity in intervention lengths, mouthwash composition, participant characteristics, and blood-pressure assessment schedules, in addition to methodological weaknesses (such as inadequate washout periods and lack of randomization) in some studies make the pooled evidence inconclusive. Consequently, no firm conclusions can be drawn regarding the effect of antibacterial mouthwash on blood pressure at this time. Future research should utilize larger, randomized trials with standard washout periods and long-term monitoring to further clarify the relationship between antibacterial mouthwash usage and blood pressure.

## Background

Oral hygiene and healthy oral microbiota are essential for overall health, locally and systemically [11]. Approximately 36% of U.S. adults use antibacterial-based mouthwash daily as part of their oral hygiene regimen [4]. Established indications for antibacterial mouthwash include plaque-induced gingivitis, post-operatively, and when patients may be unable to perform mechanical hygiene. While antibacterial mouthwashes have oral health benefits, they also eliminate beneficial oral bacteria, including *Streptococcus salivarius, Granulicatella, Veillonella,* and more [15]. These bacteria normally convert ingested nitrate into nitrite in saliva. Once swallowed, this nitrite is further reduced to nitric oxide in stomach acid and then enters systemic circulation. Nitric oxide acts as a key regulator of systolic and diastolic blood pressure by lowering peripheral vascular resistance through arteriolar vasodilation. Therefore, by eliminating these bacteria, antibacterial mouthwashes cause a downstream reduction in oral bioavailability of nitric oxide, thus increasing vascular tone and potentially raising blood pressure [14]. Given the potential cardiovascular consequences of antibacterial mouthwash usage, this scoping review aims to map the extent, range, and characteristics of the literature examining the relationship between antibacterial mouthwash use and blood pressure in humans, while identifying existing knowledge gaps.

## Methods

A scoping review methodology was chosen for this review because the small literature base, consisting of diverse methodologies, sample characteristics, interventions, and outcomes, precludes meaningful quantitative synthesis across the literature. A formal protocol for this scoping review was developed internally prior to the study, but was not prospectively registered in a public depository. To ensure transparency, the original protocol, search strategies, and data extraction forms have been made publicly available on Open Science Framework (protocol # 10.17605/OSF.IO/B29SZ). This review strictly follows the PRISMA Extension for Scoping Reviews (PRISMA-ScR) guidelines [16]. We screened the available literature using the databases PubMed and Scopus. Our search strings included keywords such as “mouthwash,” “chlorhexidine,” “antibacterial,” “salivary nitrite,” “nitrate,” and MeSH terms like “blood pressure” and “hypertension.” For example, ((“Mouthwashes”[Mesh] OR “chlorhexidine” [tiab] OR “antibacterial mouthwash” [tiab]) AND (“Blood Pressure” [Mesh] OR “Hypertension”[Mesh]) AND (2014/01/01[PDAT]: 2025/12/31[PDAT])). Inclusion criteria were: 1) analyzes changes in blood pressure (BP) after using mouthwash, 2) participants are all adult humans, 3) primary, peer-reviewed research, and 4) published from 2014–2025 (inclusive). Exclusion criteria were: 1) involving no analysis of BP change after using antibacterial mouthwash, 2) animal studies, 3) studies involving pediatric populations, 4) review articles, commentaries, expert opinions, and 5) articles published before 2014. Studies were restricted to adults because hypertension guidelines are clearer and more stringent for adults, and adult oral microbiota are more stable than pediatric oral microbiota. Grey literature was not included in our review due to failing to meet inclusion criteria (primary, peer-reviewed research). Prospective articles were not restricted by publication language. Database search results were imported into Excel, and duplicate articles were manually identified and confirmed by a reviewer. Two reviewers completed blinded title/abstract screening and full-text screening to manually select studies that met all inclusion criteria and that did not meet any exclusion criteria. Conflicts were resolved through discussion. Screening of included article reference lists was not performed, and artificial intelligence automation tools were not used during the selection process. Data extraction was completed in Excel by two reviewers. The extracted data included study methodology, sample size, sample characteristics, mouthwash type/active ingredient, length of mouthwash intervention, description of intervention regimen, and study outcomes. Discrepancies in extraction were resolved through discussion.

**Fig. 1:**
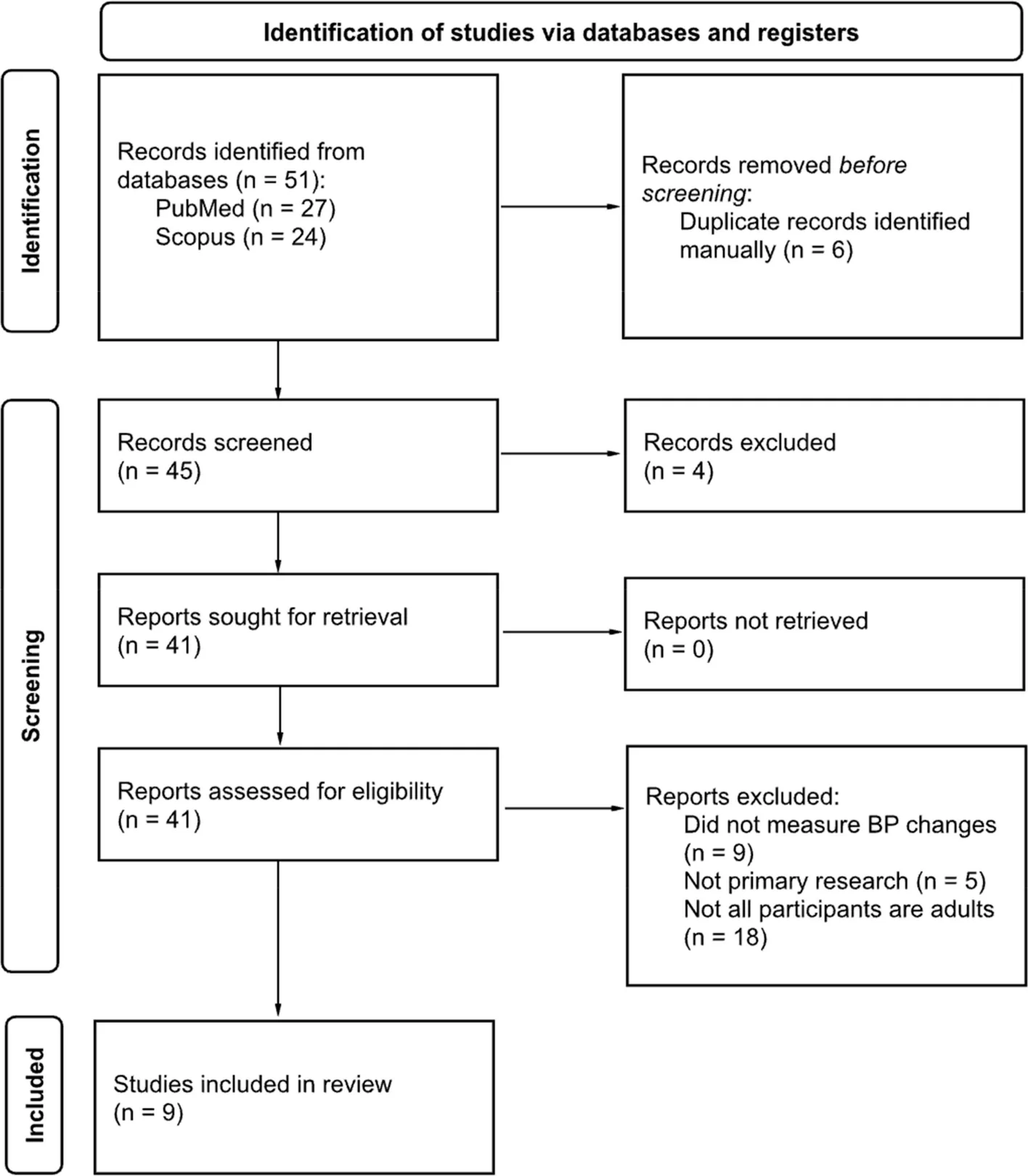
PRISMA 2020 study selection flow diagram (Page et al. 2021)

After conducting literature searches and removing duplicates, 45 records entered title/abstract screening, 41 records passed title/abstract screening, and 9 records passed full-text screening and were included in the scoping review (see Fig. 1). The most common reason for exclusion during full-text evaluation was including participants under 18 years of age.

Two reviewers independently applied the Modified Downs and Black assessment (modified for the assessment of the methodological quality of both randomized and non-randomized studies) to each included study (based on source) [10]. Disagreements between reviewers were resolved by conference.

## Results

The nine studies had a median enrollment of 30 participants and an average enrollment of 84 participants (standard deviation = 171). The sizable discrepancy between median and mean, and the large standard deviation, highlights the skewing of the mean by a large observational study and shows the variability in sample size. Due to the observational cohort disproportionately inflating the mean, the interventional trials (*n* = 10–45) are comparably underpowered to detect modest BP changes. Table 1 presents the effect direction on BP (up, down, or neutral) across all of the included studies. For a summary of study design, sample size, sample characteristics, mouthwash intervention regimen, and outcomes from each included study, see Table 2.

**Table 1:** Effect direction and methodological quality of included studies

Author	Year	Study Quality*	Significant Effect on BP
Bondonno et al.	2015	Good	↑
Woessner et al.	2016	Fair	↑
Ashworth et al.	2019	Poor	↔
Babateen et al.	2019	Good	↔
Tribble et al.	2019	Excellent	↑
Bescos et al.	2020	Good	↔
Joshipura et al.	2020	Good	↑
Aizawa et al.	2023	Fair	↑
Bescos et al.	2025	Good	↔

* Study quality further detailed in Table 3

↑ Indicates the study showed a significant effect on BP

↔ Indicates the study did not show a significant effect on BP

**Table 2:** Key extracted data from each sample

Author	Year	Study design	Sample size and characteristics	Mouthwash type/active ingredient	Mouthwash regimen	Key outcomes
Bondonno et al.	2015	Randomized crossover trial	15 treated hypertensive adults	0.12% chlorhexidine	Twice daily for 3 days	Mouthwash showed a **significant increase in systolic blood pressure (SBP) (2.3 mmHg, *p* = 0.01)** but no significant increase in diastolic blood pressure (DBP) (0.7 mmHg, *p* = 0.4).
Woessner et al.	2016	Randomized crossover trial	12 healthy, normotensive adult males	Cepacol and 0.12% chlorhexidine	One usage for each mouthwash with one week washout period between each administration.	**SBP increased significantly in Cepacol and chlorhexidine compared to control** or Listerine antiseptic (*p* < 0.05).
Ashworth et al.	2019	Nonrandomized crossover trial	Young and healthy adult vegetarians (*n* = 22) and omnivores (*n* = 19)	0.2% chlorhexidine	Twice daily for 7 days	Among all participants, **SBP and mean arterial blood pressure did not significantly increase** (*p* = 0.103) compared to the control.
Babateen et al.	2019	Randomized crossover trial	10 healthy adults	0.2% chlorhexidine	Once	**No significant difference in SBP or DBP** in mouthwash vs control (*p* > 0.05).
Tribble et al.	2019	Nonrandomized crossover trial	27 healthy normotensive adults with documented oral hygiene behaviors and free of oral disease	0.12% chlorhexidine	Twice daily for 7 days	**SBP significantly increased** with mouthwash usage compared to control (*p* < 0.04).
Bescos et al.	2020	Nonrandomized crossover trial	36 healthy adults	0.2% chlorhexidine	Twice daily for 7 days	**No significant increase in SBP (*p* = 0.052) or DBP** (*p* = 0.999) with mouthwash compared to control.
Joshipura et al.	2020	Observational study of cohort subjects	540 normotensive, obese adults enrolled in the San Juan Overweight Adults Longitudinal Study	N/A since no mouthwash intervention	N/A since no mouthwash intervention	Mouthwash usage was **significantly associated with increased likelihood of self-reported physician diagnosis of hypertension** in obese adults.
Aizawa et al.	2023	Randomized crossover trial	30 healthy older adults	“Antibacterial mouthwash”	Twice daily for 14 days	Mouthwash intervention led to a **non-significant increase in SBP** (2.0 mmHg, 95% CI [0.0, 3.9] and **significantly increased DBP** (1.2 mmHg, 95% CI [0.4, 2.0]) compared to placebo.
Bescos et al.	2025	Randomized active-controlled trial	45 healthy adults	Propolis (antibacterial) mouthwash and 0.2% chlorhexidine mouthwash	Twice daily for 7 days	There were **no significant increases in BP** after mouthwash usage compared to baseline BP (*p* > 0.05).

From the quality appraisal analysis, the mean Downs and Black score was 21.9 ± 2.9, indicating “good quality” studies (a score of 26–28 is “excellent,” 20–25 is “good,” “fair” is 15–19, and “poor” is ≤ 14) (Table 3). While the studies were overall of “good quality”, there are important weaknesses across the different studies, including the absence of blinding, low statistical power, and insufficient control of dietary nitrate intake.

**Table 3:** Modified Downs and Black quality appraisal results

Author	Year	Reporting Quality (out of 11)	External Validity (out of 3)	Internal Validity—Bias (out of 7)	Internal Validity—Confounding (out of 6)	Power (out of 1)	Total Score (out of 28)
Bondonno et al.	2015	10	2	6	5	1	24
Woessner et al.	2016	10	1	4	3	1	19
Ashworth et al.	2019	8	2	5	3	0	18
Babateen et al.	2019	10	2	6	5	0	23
Tribble et al.	2019	11	2	6	6	1	26
Bescos et al.	2020	10	2	6	5	1	24
Joshipura et al.	2020	10	2	6	5	1	24
Aizawa et al.	2023	10	1	4	3	1	19
Bescos et al.	2025	9	1	6	3	1	20

Bondonno et al. [8] performed a randomized crossover trial of 15 treated hypertensive adults. Subjects had their BP measured at the end of their three-day period of using the intervention of 0.12% chlorhexidine twice daily and at the end of their three-day period of the plain water rinse intervention twice daily. Subjects were randomly assigned to the intervention they would complete first, and the three-day periods were separated by 10–12 washout days. The comparison of BP readings indicated that mean systolic blood pressure (SBP) increased significantly (2.3 mmHg, *P* = 0.01) after the mouthwash period, when compared with after the tap water period. However, the difference in mean diastolic blood pressure (DBP) was not significant (0.7 mmHg, *P* = 0.4).

Woessner et al. [20] conducted a randomized crossover trial of 12 normotensive adult males using three different mouthwashes: Listerine (active ingredients: eucalyptol, menthol, methyl salicylate, thymol), Cepacol (cetylpyridinium chloride), and 0.12% chlorhexidine. At each of four visits, subjects had a baseline BP check, consumed 140 mL of beetroot juice (a source of 8.4 mmol of nitrates), waited 15 min, used one of three mouthwashes or the control (water), and then had BP checked every hour for four hours. SBP increased significantly after using Cepacol and chlorhexidine compared to control or Listerine antiseptic (*P* < 0.05). The authors concluded that “stronger” mouthwashes, such as Cepacol and chlorhexidine, were more likely to cause elevated SBP.

Ashworth et al. [2] recruited 41 young, healthy adults to use the placebo mouthwash twice daily for one week and 0.2% chlorhexidine mouthwash twice daily for one week. BP was measured weekly, and SBP and mean arterial BP did not significantly increase (*P* = 0.103) after completing the chlorhexidine mouthwash regimen compared with after the placebo regimen.

Similar to Woessner et al., Babateen et al. [3] analyzed the effect of one use of mouthwash on BP. They recruited 10 healthy adults to complete a randomized crossover trial composed of two visits. At each visit, baseline vitals were obtained from participants, who were then instructed to rinse their mouth with 20 mL of low-nitrate water or 20 mL of 0.2% chlorhexidine mouthwash for two minutes. 15 min later, they were given 70 mL of beet juice (~ 6.5 mmol of nitrate). Saliva and BP readings were collected hourly for the next five hours. Participants attended their second visit 24 h later and completed the same steps but received the other intervention (20 mL of low-nitrate water or 20 mL of 0.2% chlorhexidine mouthwash). Babateen et al. found no significant difference in SBP or DBP after chlorhexidine compared to water (*P* > 0.05).

Tribble et al. [19] performed a non-randomized crossover trial of 27 healthy adults. All participants used 0.12% chlorhexidine mouthwash twice daily for 7 days, then discontinued it for seven days. BP was measured at study onset, day 7, day 10, and day 14. The authors found that SBP significantly increased after seven days of twice-daily chlorhexidine mouthwash compared with baseline SBP (*p* < 0.04). SBP and DBP significantly decreased on the third day after discontinuing chlorhexidine (*p* < 0.04).

Bescos et al. [6] recruited 36 healthy adults for their non-randomized crossover trial. Similar to Ashworth et al., participants used the placebo mouthwash twice daily for one week, followed by 0.2% chlorhexidine mouthwash twice daily for one week, with BP measurements taken after each week. Comparing BP at completion of the chlorhexidine regimen to BP at completion of the placebo regimen, Bescos et al. [6] found that SBP increased, but not significantly (*P* = 0.052), and DBP did not change significantly (*P* = 0.999).

Contrary to the other articles in this review, Joshipura et al. [13] is the only observational study. Using the San Juan [Puerto Rico] Overweight Adults Longitudinal Study, they enrolled all normotensive cohort members and followed up three years later, asking them about their mouthwash usage and whether they had been diagnosed with hypertension by a medical professional. They found that mouthwash usage was significantly associated with hypertension in obese adults (*P* < 0.05). Furthermore, the relationship was stable across all analyzed sub-groups, and it was especially strong among groups with low hypertension risk factors (never smoked, younger adults, meeting exercise recommendations, etc.).

Aizawa et al. [1] conducted a randomized crossover trial of 30 healthy older adults in two phases. In the first phase, participants were randomized to either a two-week active nitrate intervention of 12.4 mmol nitrate in beetroot juice daily or a two-week placebo. There was a two-week washout period followed by the subjects completing the other intervention for two weeks and then another two-week washout. In the second phase, subjects used an unspecified antibacterial mouthwash intervention twice daily for two weeks. BP was measured at the beginning and at the end of each of the two-week periods. Comparing BP at the end of the mouthwash intervention to the end of the placebo intervention, SBP increased but not significantly (2.0 mmHg, 95% CI [0.0, 3.9]), but DBP increased significantly (1.2 mmHg, 95% CI [0.4, 2.0]).

In a subsequent 2025 study, Bescos et al. [7] analyzed the effect of 0.2% chlorhexidine mouthwash and propolis mouthwash on BP. Propolis is a traditional antibacterial medicine therapy derived from honeybee resin [5]. In the study, 45 healthy adult participants were randomized to use propolis or chlorhexidine twice daily for one week. BP was measured at the onset and at the end of its one-week period. There were no significant increases in BP after one week of twice-daily usage of propolis or chlorhexidine compared to baseline BP (*P* > 0.05).

## Discussion

Dietary nitrate varies tremendously with the source, and it is associated with both cardiovascular benefits and increased cancer risk, though this may depend on the food source (plants v. water and processed meats) [9, 12]. Furthermore, there is increasing evidence of the interaction between the oral and gut microbiota, which may have very significant effects on human health [17]. While other studies of antibacterials focus on the relationship between gut microbiome and cardiovascular health [18], here we examine the connection between oral microbiota and cardiovascular health. In this scoping review, we identified nine studies that investigated the relationship between the use of antibacterial mouthwash and BP in humans. Five studies found a significant increase in SBP or DBP with antibacterial mouthwash use (presence of at least one significant relationship between mouthwash and BP), and four found no significant relationship in SBP or DBP with mouthwash use (Table 1). Even when statistically significant, the observed SBP elevations (e.g., 2.3 mmHg in Bondonno et al.) were small. The heterogeneity of results warrants investigation into the different intervention regimens, participant characteristics, and methodologies across the literature.

Mouthwash intervention length varied across the included studies, since two studies used the mouthwash once, one study used the mouthwash for three days, and six studies used the mouthwash for seven days or more. The most common regimen was twice daily for seven days (four studies). All experimental studies with multiple uses of mouthwash instructed participants to use it twice daily. Despite the different intervention lengths, there are no discernible differences in the outcomes between different experimental intervention regimens. Of the six studies that used mouthwash for seven or more days, three found a significant effect on SBP or DBP, and three found no significant effect. Similarly, the two studies that administered mouthwash once were split on the significance of the effect of mouthwash on BP. However, the outlier in mouthwash usage involved the observational study by Joshipura et al. [13], which noted that mouthwash use over three years was associated not only with increased SBP and DBP but also with a diagnosis of hypertension. It was the only study to find clinically significant increases in BP. There are certainly limitations with this study (observational design limiting inferences, self-report mouthwash usage and self-reported hypertension can involve bias, and the sample of obese adults limited generalizability), and there are no other studies of a similar length of time to compare to, but this finding may indicate that antibacterial mouthwash usage could affect BP cumulatively through long-term usage, and this effect would be missed or barely noticeable when only using mouthwash for one administration or 7–14 days. However, Joshipura et al. was the only study to examine mouthwash usage longer than a few weeks, so the long-term cardiovascular implications of antibacterial mouthwash use remain uncertain.

In addition to mouthwash regimen, type of mouthwash varied across the included articles. Four studies used 0.2% chlorhexidine, three studies included 0.12% chlorhexidine, one study used Cepacol (active ingredient: 0.05% cetylpyridinium chloride), and one study used propolis. All three studies that used 0.12% chlorhexidine found a significant increase in BP, but, interestingly, all four studies with 0.2% chlorhexidine found no significant increase in BP. One could interpret this to show that there is not a dose–response relationship between antibacterial mouthwash usage and BP. This hypothesis is further supported by our earlier observation that intervention length did not appear to affect the relationship between antibacterial mouthwash and BP. However, 0.12% and 0.2% chlorhexidine were not compared within the same study, so it would be premature to draw conclusions regarding the effects of different doses of chlorhexidine on BP with the current state of the literature. Furthermore, Cepacol had a significant effect on BP, but propolis had no significant effect, however each was only examined in one study, so we refrain from forming conclusions about the effects of Cepacol or propolis on BP.

The selected literature also varied in the participant characteristics. The vast majority of studies sampled healthy adults (*n* = 7); their results were mixed, with four having had no significant relationship between mouthwash and BP, and three finding a significant relationship. Among the remaining two studies, one sampled adults with controlled hypertension and the other sampled obese adults, and both found that mouthwash had a significant effect on BP. This finding is supported by a meta-analysis of mouthwash usage among adults with mild hypertension, which found mouthwash usage to slightly but significantly increase the risk of developing hypertension [4].

An interesting finding from the observational study of obese adults is that while the relationship between mouthwash usage and hypertension was significant across all subgroups, Joshipura et al. [13] found that the association was especially strong among obese adults with lower risk factors for hypertension. This indicates that the effect of mouthwash on elevated BP may be obscured by other hypertension risk factors. This would seem to contradict the studies of healthy adults (as we would expect most of these studies to find a significant relationship between mouthwash usage and elevated BP). However, one possible resolution is hypothesizing that healthy individuals are better able to regulate BP, so the effect of mouthwash on BP is attenuated, while obese individuals have weaker BP regulation, potentially making the effect of mouthwash stronger, contributing to the expression of hypertension.

Notably, there were four distinct methodologies in our scoping review literature: randomized crossover trials (*n* = 4), nonrandomized crossover trials (*n* = 3), a randomized active-controlled trial (*n* = 1), and an observational study (*n* = 1). The crossover design was the predominant study design because subjects function as their own controls (as long as there is a sufficient washout period). However, there is no consensus in the literature regarding sufficient washout period length, as noted by Bescos et al. [6], and this was the justification the three studies provided for choosing a nonrandomized design. Two out of three nonrandomized crossover trials found no significant effect, but three out of four randomized crossover trials found mouthwash usage to have a significant increase in SBP or DBP. Therefore, within the emerging literature, randomized crossover trials might better capture BP changes due to mouthwash, but the small sample of studies within this review heavily limits this inference. Furthermore, it would be difficult to conduct randomized crossover trials over multiple years to evaluate the long-term effects of antibacterial mouthwash that were identified in Joshipura et al. [13].

There are some important limitations to our study. The chief limitation is the small sample sizes of the studies, limiting their power. Additionally, the low participant counts, combined with modest effect sizes, increase the likelihood of type I and type II errors. While all trial studies with multiple administrations of mouthwash used a twice-daily schedule, all relied on participants’ self-reported adherence to the mouthwash schedule. Some studies used narrow participant characteristics (i.e. adults with hypertension, males, obese adults) which limits the external validity of each study, comparison across studies, and the generalizability of our review. Comparability was further hampered due to variation in the literature in the amount of mouthwash used, type of mouthwash, mouthwash concentration, length of washout periods, description (or lack thereof) of participant oral health, and timing for BP measurements. Additionally, many studies measured BP as a secondary outcome, so there was less detailed reporting on the BP data. Other limitations of our study were searching only two databases (PubMed and Scopus) and the lack of grey literature. Grey literature did not meet the inclusion criterion for our peer review process. However, it is an important part of the literature landscape and can be included in future work to highlight conceptual endeavors. Lastly, our Downs and Black analysis (Table 3) showed that our studies were of “good quality,” however, that does not preclude studies in our analysis from having important weaknesses in generalizability, power, and control of confounding variables. Given these limitations, the low number of studies in our review, and significant variation between studies, there is insufficient data to draw firm conclusions on the relationship between antibacterial mouthwash and BP. Despite these limitations, there are important strengths to our review, including adherence to standardized PRISMA-ScR guidelines, public availability of the protocol on Open Science Framework, Downs and Black quality assessment, and providing a synthesis of an emerging field of research.

## Conclusion

The objective of this scoping review was to evaluate the evidence for antibacterial mouthwash altering BP in humans. We determined that the limited available evidence is mixed, with some short-term studies showing statistically significant, but not clinically significant, BP increases and some showing no significant changes, and substantial variation between studies hampering clear takeaways. While the one long-term study in our review found a significant relationship between mouthwash usage and hypertension, it is subject to self-report and recall bias, and it only examined obese adults (while most other studies in our review examined healthy adults). These findings highlight the need for standardized research.

Future studies should prioritize evaluating the long-term cardiovascular effects of antibacterial mouthwash, ideally through multi-year prospective cohort studies or pragmatic clinical trials. Standardization of mouthwash concentration, dosing frequency, and washout periods is essential to improve comparability across studies. Additionally, further research should incorporate controlled dietary nitrate intake, detailed microbiome profiling, and measurement of salivary nitrate in order to clarify mechanistic pathways. Antibacterial mouthwashes are often indicated for patients with poor periodontal health or post-surgical oral pathologies, yet many studies examined healthy adults. Future work could study populations most likely to be prescribed antibacterial mouthwashes or provide strict controls of oral health parameters. Furthermore, including other diverse populations, such as individuals with hypertension, metabolic disease, or impaired endothelial dysfunction, may help identify subgroups most susceptible to BP changes. Lastly, the effects of other antibacterial oral health products (such as antibacterial toothpastes or antibacterial dental floss) on BP should also be investigated.

Given that 36% of U.S. adults use mouthwash daily [4], understanding whether antibacterial mouthwash contributes to elevated BP is an important public health priority. While the data presented here do not demonstrate a significant link between use of mouthwash and development of hypertension, a long-term pathology, further studies are indicated to determine if there is such a link. As the incidence of hypertension continues to increase world-wide, understanding possible modifiers is a necessity. With this knowledge, patients, healthcare professionals, and policymakers can make informed decisions regarding the use and promotion of antibacterial mouthwash rinses, tailored to patient population needs.

## Data Availability

Not applicable as this is a secondary review of published articles.

## Abbreviations

BP: Blood pressure
SBP: Systolic blood pressure
DBP: Diastolic blood pressure
U.S.: United States
